# Nomograms to predict long‐term survival for patients with gallbladder carcinoma after resection

**DOI:** 10.1002/cnr2.1991

**Published:** 2024-03-05

**Authors:** Shilei Bai, Pinghua Yang, Jiliang Qiu, Jie Wang, Liu Liu, Chunyan Wang, Huifeng Wang, Zhijian Wen, Baohua Zhang

**Affiliations:** ^1^ Department of Hepatic Surgery II The Eastern Hepatobiliary Surgery Hospital, Naval Medical University Shanghai China; ^2^ Department of Biliary Surgery IV The Eastern Hepatobiliary Surgery Hospital, Naval Medical University Shanghai China; ^3^ Department of Hepatobiliary Surgery Sun Yat‐Sen University Cancer Center Guangzhou China; ^4^ State Key Laboratory of Oncology in South China and Collaborative Innovation Center for Cancer Medicine Sun Yat‐Sen University Guangzhou China; ^5^ Department of Hepatic Surgery The Fifth Clinical Medical College of Henan University of Chinese Medicine; ^6^ Department of Hepatobiliary Pancreatic Vascular Surgery The Chenggong Hospital, Xiamen University Xiamen China

**Keywords:** decision curve analysis, gallbladder carcinoma, nomogram, prognosis, resection, TNM system

## Abstract

**Background:**

Surgical resection remains the primary treatment option for gallbladder carcinoma (GBC). However, there is a pressing demand for prognostic tools that can refine patients' treatment choices and tailor personalized therapies accordingly.

**Aims:**

The nomograms were constructed using the data of a training cohort (*n* = 378) of GBC patients at Eastern Hepatobiliary Surgery Hospital (EHBH) between 2008 and 2018. The model's performance was validated in GBC patients (*n* = 108) at Guangzhou Centre from 2007 to 2018.

**Methods and results:**

The 5‐year overall survival (OS) rate in the training cohort was 24.4%. Multivariate analyses were performed using preoperative and postoperative data to identify independent predictors of OS. These predictors were then incorporated into preoperative and postoperative nomograms, respectively. The C‐index of the preoperative nomogram was 0.661 (95% CI, 0.627 to 0.694) for OS prediction and correctly delineated four subgroups (5‐year OS rates: 48.1%, 19.0%, 15.6%, and 8.1%, *p* < 0.001). The C‐index of the postoperative nomogram was 0.778 (95%CI, 0.756 –0.800). Furthermore, this nomogram was superior to the 8th TNM system in both C‐index and the net benefit on decision curve analysis. The results were externally validated.

**Conclusion:**

The two nomograms showed an optimally prognostic prediction in GBC patients after curative‐intent resection.

## 1 INTRODUCTION

Gallbladder carcinoma (GBC) is a frequent biliary tract neoplasm and ranks sixth in terms of occurrence among digestive system malignancies.^1^ Despite advancements in therapeutic approaches including surgery, (neo)adjuvant therapies, radiotherapy, and immunotherapies, GBC's prognosis remains grim, with a five‐year survival rate of less than 5%.^2^ Surgical resection stands as the primary treatment for GBC. Unfortunately, the long‐term outcomes following resection remain unsatisfactory due to rapid progression.^3^ Consequently, there's an immediate need for prognostic tools to enhance the optimization of patients' treatment options, tailored to their specific needs. Notably, a precise prediction model for overall survival (OS) could prove instrumental in identifying high‐risk subgroups with more unfavorable prognoses, enabling targeted treatment strategies post‐resection.

The commonly employed staging system for gallbladder carcinoma (GBC) is the American Joint Committee on Cancer (AJCC) 8th staging system.^4^ This system is instrumental in customizing (neo)adjuvant therapies, predicting postsurgical prognosis, and guiding additional adjuvant treatment. However, the simplification process during the creation of this system could have led to the exclusion of several pivotal prognostic factors, including resection margin, tumor differentiation, and perineural invasion.^5^ As a result, the potential for effective prognostic stratification in the context of GBC remains subject to debate.

Prognostic nomograms have demonstrated their effectiveness in numerous malignancies, surpassing the predictive potential of conventional tumor staging systems.^6^, ^7^, ^8^ In fact, nomograms possess the unique capability to generate personalized predictions, making them an appealing substitute or alternative standard to the AJCC staging system.^9^ A notable example is the study conducted by Wang et al, which crafted a predictive nomogram model tailored to the implementation of adjuvant radiotherapy in a cohort of 4180 GBC patients.^10^ However, it's worth noting that this model lacked the inclusion of surgical margin status and detailed information about the extent of surgical resection, possibly limiting its applicability.^11^, ^12^ Given this context, the development of more comprehensive and precise nomograms holds promise in enhancing predictive accuracy for GBC patients who have undergone curative‐intent resection.

In this study, we formulated a pair of prognostic nomograms tailored to GBC patients who have undergone curative‐intent resection, integrating both pre‐ and postoperative data. The first nomogram serves as a valuable tool for guiding the selection of surgical treatment, while the second nomogram holds the potential for accurately forecasting individual postoperative outcomes.

## 2 MATERIALS AND METHODS

### 2.1 Patients and study design

We conducted this study at the Eastern Hepatobiliary Surgery Hospital in Shanghai, enrolling consecutive patients who had undergone curative‐intent resection for pathologically confirmed GBC between January 2008 and December 2018. The data of these patients were collected prospectively. The inclusion criteria consisted of the following: (a) preoperative performance status score of 0–1; (b) absence of distant metastasis; (c) no previous history of other carcinomas; d. no prior anti‐tumor therapy before surgery. The exclusion criteria encompassed cases with macroscopically positive (R2) surgical margins, insufficient clinical information, and patients lost to follow‐up. All eligible patients undergoing resection at the Shanghai center were included in the training cohort. For external validation, a separate cohort of consecutive patients at the Sun Yat‐Sen University Cancer Center in Guangzhou, between February 2007 and December 2018, was established using the same selection criteria. Ethical approval was granted by the Institutional Ethics Committee of the hospital, and all patients provided informed consent for their data to be collected for the study's purposes.

Both centers shared similar indications for resection and perioperative management. Preoperative evaluations encompassed liver function tests, routine blood panels, prothrombin time assessments, carcinoembryonic antigen (CEA), and carbohydrate antigen 19‐9 (CA19‐9) tests. Imaging studies included chest radiography, abdominal ultrasonography, and contrast‐enhanced computed tomography (CT)/magnetic resonance imaging (MRI) of the abdomen. Comprehensive assessments of cardio‐pulmonary function were conducted for elderly patients or those with significant co‐existing conditions. For some jaundiced patients, percutaneous transhepatic cholangiography drainage (PTCD) or endoscopic retrograde cholangio‐pancreatography (ERCP) was typically performed approximately a week before hepatectomy if the re‐evaluation of hepatic function was deemed necessary. The clinical staging was determined based on the pathological tumor‐node‐metastasis (pTNM) classification in accordance with the 8th edition guidelines of the American Joint Committee on Cancer (AJCC).^4^

### 2.2 Operative procedures

The surgical approach followed the previously reported strategy.^13^ Radical surgery encompassed en bloc resection of the gallbladder, partial resection into the liver, and dissection of regional lymph nodes. Partial hepatectomy referred to the removal of a wedge with a 2 cm margin (including segments IVb/V), extended right/left hepatectomy, or right trisectionectomy. Regional lymph nodes included those situated on the hepatoduodenal ligament, hepatic artery, and those located behind the pancreatic head. When adjacent organs (such as the bile duct, stomach, duodenum, colon, omentum, and pancreatic head) were invaded, they were also resected to achieve an R0 resection. In this context, R0 resection indicated pathologically negative transactional margins, R1 resection denoted the presence of microscopically evident residual disease (positive margins), and R2 resection signified the presence of gross (macroscopic) residual disease^14^ Lymph metastasis was defined as N1 for one to three positive nodes and N2 for four or more positive nodes, as stipulated in the AJCC staging system (8th edition).

### 2.3 Follow‐up

The established follow‐up protocol encompassed regular physical examinations and laboratory assessments, which included monitoring CEA and CA19‐9 levels. These evaluations were conducted every 3 months during the initial 2 years and shifted to a 6‐month interval for the subsequent 3 years. In cases where CA 19‐9 levels were elevated or signs of potential recurrence were observed, enhanced CT, MRI, or PET‐CT scans were promptly conducted. Depending on the circumstances at the time of recurrence, interventions such as re‐operation, (neo)adjuvant therapy, radiotherapy, or traditional Chinese medicine treatment were employed.

### 2.4 Statistical analysis

OS was defined as the duration spanning from the surgical procedure to either the patient's demise or the last follow‐up. Continuous variables with a normal distribution were subjected to Student's *t*‐test, while the Mann–Whitney *U* test was employed for non‐normally distributed variables. Categorical variables were analyzed using the *χ* ^2^ test or Fisher's exact test when suitable. The Kaplan–Meier method was employed to construct survival curves, and the log‐rank test was used for their comparison. Multivariate analyses were conducted using Cox proportional analysis with backward stepwise selection based on the Akaike information criterion.^15^ Nomograms predicting OS were devised according to the outcomes of multivariate analyses. The predictive nomogram's efficacy was gauged using the concordance index (C‐index), while predictions were illustrated through Kaplan–Meier curves for quartiles and calibration plots. Model validation utilized 1000 resampling bootstraps.^16^ The performance of the nomograms was assessed via decision curve analysis (DCA), a procedure outlined in an accessible online tutorial.^17^, ^18^ Statistical computations were executed using R 2.13.2 (http://www.r-project.org/). *p*‐Values less than .05 were deemed statistically significant.

## 3 RESULTS

### 3.1 Clinicopathological features and post‐resection OS

A total of 429 consecutive GBC patients who underwent curative intent resection were initially identified. Among them, 61 were excluded due to factors including preoperative adjuvant chemoradiotherapy (*n* = 13), metastatic disease (*n* = 3), R2 surgical margins (*n* = 23), history of other malignancies (*n* = 4), perioperative death (*n* = 10), and early loss to follow‐up after discharge (*n* = 8). The training cohort was composed of the remaining 378 patients. In the external validation cohort, 22 out of 130 patients were excluded due to preoperative adjuvant chemoradiotherapy (*n* = 4), R2 surgical margins (*n* = 10), perioperative death (*n* = 3), and loss to follow‐up (*n* = 5). The remaining 108 patients constituted the external validation cohort.

The baseline clinicopathological characteristics in the cohorts exhibited general similarity. Yet, within the training cohort, the percentage of GBC patients with preoperative serum CA 19‐9 levels >37 (67.8% vs. 42.6%) was notably higher compared to the external validation cohort from the Guangzhou Centre (Table 1). The performed types of resection in GBC patients are outlined in Supplemental Table 1, and the associated surgical complications are detailed in Supplemental Table 2.

**TABLE 1:** Baseline characteristics of patients between training cohort and validation cohort.

	Number (%)/Median (IQR)			*p*‐Value
Variable	ALL (*n* = 486)	Training cohort (*n* = 378)	Validation cohort (*n* = 108)	
Age, years	59.0 (53.0–66.0)	60.0 (53.0–66.0)	57.0 (49.0–65.2)	.208
Sex				
Female	304 (62.7%)	234 (62.1%)	70 (64.8%)	.582
Male	182 (37.3%)	144 (37.9%)	38 (35.2%)	
Hypertension				
Absence	394 (81.1%)	303 (80.2%)	91 (84.3%)	.337
Presence	92 (18.9%)	75 (19.8%)	17 (15.7%)	
Diabetes mellitus				
Absence	453 (93.2%)	355 (93.9%)	98 (90.7%)	.248
Presence	33 (6.8%)	23 (6.1%)	10 (9.3%)	
Body mass index, kg/m^2^	23.1 (20.9–24.9)	23.1 (21.1–24.9)	22.8 (20.5–24.8)	.351
Associated gallbladder disease				
Absence	223 (45.9%)	169 (44.7%)	54 (50.0%)	.330
Presence	263 (54.1%)	209 (55.3%)	54 (50.0%)	
Jaundice				
Absence	371 (76.3%)	285 (75.4%)	86 (79.6%)	.371
Presence	115 (23.7%)	93 (24.6%)	22 (20.4%)	
TBIL, μmol/L	13.1 (9.33–24.8)	12.9 (8.83–27.1)	15.4 (11.8–20.7)	.067
ALT, U/L	29.3 (17.6–69.9)	29.3 (16.0–78.0)	29.4 (22.5–57.9)	.237
CEA, ng/mL	3.00 (1.60–9.07)	2.90 (1.60–8.88)	4.10 (1.60–10.0)	.265
CA 19–9, U/mL	111 (16.4–385)	132 (19.9–524)	31.0 (15.2–211)	.001
≤37	184 (37.4%)	122 (32.2%)	62 (57.4%)	
37–1000	232 (47.5%)	195 (51.3%)	37 (34.2%)	
≥1000	70 (15.1%)	61 (16.5%)	9 (8.4v)	
Abnormal lymph node (I)^a^				.449
No	415 (83.4%)	316 (83.6%)	99 (82.5%)	
Yes	81 (16.6%)	62 (16.4%)	19 (17.5%)	
Liver or/and adjacent organ invasion (I)				
Absence	287 (59.1%)	223 (59.0%)	64 (59.3%)	.961
Presence	199 (40.9%)	155 (41.0%)	44 (40.7%)	
Tumor location (I)				.170
Neck	138 (28.8%)	113 (29.8%)	25 (25.0%)	
Fundus or body	348 (71.2%)	265 (70.2%)	83 (75.%)	
Extrahepatic bile duct resection				
No	357 (73.5%)	281 (74.3%)	76 (70.4%)	.410
Yes	129 (26.5%)	97 (25.7%)	32 (29.6%)	
Gallbladder resection only				
No	462 (95.1%)	359 (95.0%)	103 (95.4%)	.867
Yes	24 (4.9%)	19 (5.0%)	5 (4.6%)	
Intraoperative blood transfusion				
No	405 (83.3%)	320 (84.7%)	85 (78.7%)	.143
Yes	81 (16.7%)	58 (15.3%)	23 (21.3%)	
Resection				
R0	322 (66.3%)	253 (66.9%)	69 (63.9%)	.555
R1	164 (33.7%)	125 (33.1%)	39 (36.1%)	
Tumor location (P)^a^				
Neck	127 (26.1%)	102 (27.0%)	25 (23.1%)	.424
Fundus or body	359 (73.9%)	276 (73.0%)	83 (76.9%)	
Tumor differentiation				
Well or moderate	390 (80.2%)	308 (81.5%)	82 (75.9%)	.201
Poor	96 (19.8%)	70 (18.5%)	26 (24.1%)	
pT category^b^				.070
T1	24 (4.9%)	19 (5.0%)	5 (4.6%)	
T2	42 (8.6%)	28 (7.4%)	14 (12.9%)	
T3	354 (72.8%)	285 (75.3%)	69 (63.8%)	
T4	66 (13.7%)	46 (12.3%)	20 (v%)	
pN category^b^				.224
N0	271 (55.7%)	203 (53.7%)	68 (62.9%)	
N1	185 (38.0%)	150 (39.6%)	35 (32.4%)	
N2	30 (6.3%)	25 (6.3%)	5 (4.9%)	
AJCC stage 8th				
I	24 (4.9%)	19 (5.0%)	5 (4.6%)	.462
II	45 (9.2%)	33 (8.7%)	12 (11.1%)	
III	324 (66.6%)	256 (67.7%)	68 (62.9%)	
IV	93 (19.3%)	70 (18.6%)	23 (21.4%)	

Abbreviations: ALT, alanine aminotransferase; CA19‐9, cancer antigen 19‐9; CEA, carcinoembryonic antigen; GBC, gallbladder carcinoma; IQR, interquartile range; TBIL, total bilirubin.

^a^ (I), imaging studies; (P), postoperative pathological examinations.

^b^ pT category and pN category were defined according to the 8th edition of the AJCC staging system.

The median OS duration for the training cohort was 22.2 months and for the validation cohort, it was 23.5 months. The 1‐, 3‐, and 5‐year OS rates for the training cohort were 68.5%, 42.0%, and 24.4%, respectively, and for the validation cohort, these rates were 58.1%, 34.2%, and 26.4%, respectively. These calculations were based on data until May 15, 2022. Notably, no significant disparities in the 1‐, 3‐, and 5‐year OS rates were observed between the two cohorts (*p* = .543).

### 3.2 Independent risk factors and construction of the OS nomogram in the training cohort

The univariate analysis of OS based on the pre‐operative data is presented in Supplemental Table 3. The multivariate analysis revealed several significant factors associated with OS. Increasing CA19‐9 levels (37–1000 U/mL: HR: 1.161, 95% CI: 0.879–1.533, *p* = .293; >1000 U/mL: HR: 1.639, 95% CI: 1.146–2.343, *p* = .007), invasion of the liver or adjacent organs (HR: 1.713, 95% CI: 1.347–2.179, *p* < .001), presence of lymph nodal abnormalities (HR: 1.952, 95% CI: 1.451–1.903, *p* < .001), and tumor location (HR: 1.446, 95% CI: 1.099–1.903, *p* = .008) based on imaging were identified as independent risk factors for OS, as shown in Table 2.

**TABLE 2:** Multivariable analysis of OS based on the preoperative data in the training cohort.

	OS			
Variables	HR	95% CI		*p*‐Value
CA19‐9, U/mL				
37–1000 vs. ≤37	1.161	0.879	1.533	.293
≥1000 vs. ≤37	1.639	1.146	2.343	.007
Liver or/and adjacent organ invasion (yes: no)	1.713	1.347	2.179	<.001
Abnormal lymph node(I)^a^ (yes: no)	1.952	1.451	1.903	<.001
Tumor location (I) (neck: fundus or body)	1.446	1099	1.903	.008

Abbreviations: CA19‐9: cancer antigen 19–9; CI, confidence interval; HR, hazard ratio.

^a^ (I), imaging studies.

The univariate analysis of OS based on the post‐operative data is also presented in Supplemental Table 4. The results of the multivariate analysis indicated that higher CA19‐9 levels (37–1000 U/mL: HR: 1.419, CI: 1.076–1.871, *p* = .013; >1000 U/mL: HR: 1.617, 95% CI: 1.126–2.323, *p* = .009), R1 resection (HR: 2.938, 95% CI: 2.199–3.925, *p* < .001), tumor location in the neck (HR: 1.662, 95% CI: 1.233–2.241, *p* = .001), poor tumor differentiation (HR: 1.406, 95% CI: 1.038–1.903, *p* = .028), pT category (T2: HR: 1.348, 95% CI: 0.808–2.249, *p* = .252; T3: HR: 2.122, 95%CI: 1.290–3.490, *p* = .003; T4: HR: 3.181, 95% CI: 1.652–6.127, *p* = .001), and pN category (N1: HR: 1.824, 95% CI: 1.350–2.465, *p* = .001; N2: HR: 3.801, 95% CI: 2.387–6.053, *p* < .001) were identified as independent risk factors for OS, as summarized in Table 3.

**TABLE 3:** Multivariable analysis of OS based on the postoperative data in the training cohort.

	OS			
Variables	HR	95% CI		*p*‐Value	
CA19‐9, U/mL				
37–1000 vs. ≤37	1.419	1.076	1.871	.013
≥1000 vs. ≤37	1.617	1.126	2.323	.009
Tumor location(P)^a^ (neck: fundus or body)	1.662	1.233	2.241	.001
Resection (R1: R0)	2.938	2.199	3.925	<.001
pT category^b^				
T2 vs. T1	1.348	0.808	2.249	.252
T3 vs. T1	2.122	1.290	3.490	.003
T4 vs. T1	3.181	1.652	6.127	.001
pN category^b^				
N1 vs. N0	1.824	1.350	2.465	.001
N2 vs. N0	3.801	2.387	6.053	<.001
Tumor differentiation (poor: well or moderate)	1.406	1.038	1.903	.028

^a^ CA19‐9, cancer antigen 19‐9; (P), postoperative pathological examinations.

^b^ pT category and pN category were defined according to the 8th edition of the AJCC staging system.

### 3.3 Nomogram performance in the training cohort

**FIGURE 1:**
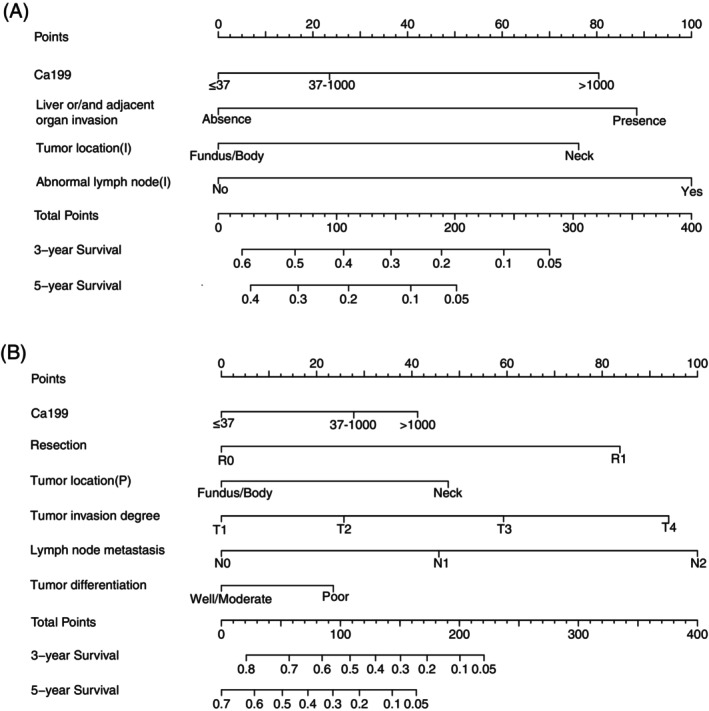
Nomogram for predicting the OS of gallbladder carcinoma patients after resection. The individual patient's value is initially identified on the variable axis and a vertical line is drawn from the value to identify the number of points for the variable value. The sum of these is found on the Total Points axis and a downward line from this value indicates the position of the predicted 3 or 5‐year survival rate. (A) The pre‐operative nomogram. (B) The post‐operative nomogram.

**FIGURE 2:**
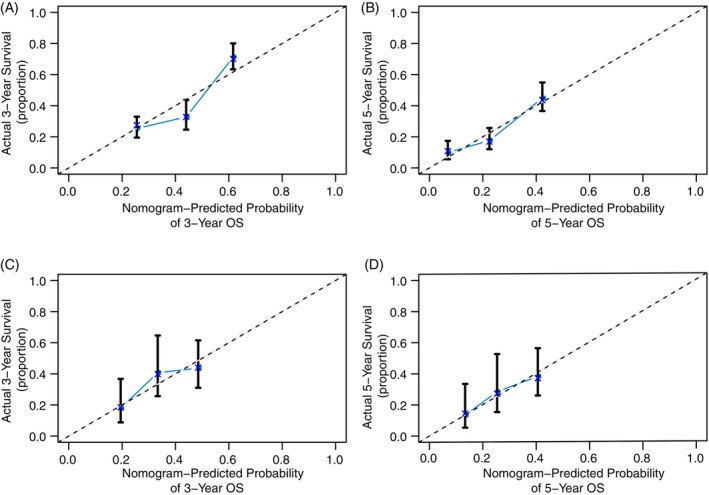
Calibration curves for OS prediction by the pre‐operative nomogram. (A and B): 3‐ and 5‐ year postoperative OS in the training cohort. (C and D) E: 3‐ and 5‐ year postoperative OS in the external validation cohort. The *x* axis represents the nomogram prediction; the *y* axis indicates the actual OS.

The preoperative nomogram, which integrates all the identified independent risk factors for OS in the training cohort, is depicted in Figure 1A. The bootstrap‐corrected C‐index for predicting OS was calculated as 0.661 (95% CI, 0.627 to 0.694). Notably, the calibration plot for the OS probability at 3 and 5 years post‐surgery (Figure 2A,B) illustrates an excellent alignment between the nomogram predictions and the actual observed data. Furthermore, the OS nomogram effectively stratified patients into four distinct prognostic subgroups. These subgroups exhibited 5‐year OS rates of 48.1%, 19.0%, 15.6%, and 8.1%, respectively (*p* < .001, Supplemental Figure 1A).

**FIGURE 3:**
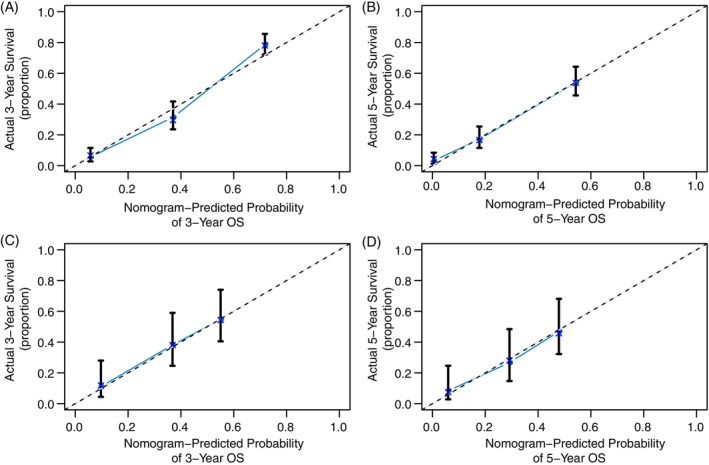
Calibration curves for OS prediction by the post‐operative nomogram. (A and B): 3‐ and 5‐ year postoperative OS in the training cohort. (C and D): 3‐ and 5‐ year postoperative OS in the external validation cohort. The *x*‐axis represents the nomogram prediction; the *y* axis indicates the actual OS.

The postoperative nomogram, which encompasses all the independent risk factors for OS identified in the training cohort, is presented in Figure 1B. The bootstrap‐corrected C‐index for predicting OS was determined as 0.778 (95% CI: 0.756–0.800). The calibration curves, after bootstrap correction, for the nomogram's ability to predict the probability of OS at 3 or 5 years following surgery exhibited favorable fitting (Figure 3A,B). Notably, the subgroups generated by the nomogram showed distinct 5‐year OS rates of 57.2%, 25.9%, 7.8%, and 1.0%, respectively (*p* < .001, Supplemental Figure 1B). Furthermore, the OS nomogram's C‐index surpassed that of the 8th TNM system (0.778 vs. 0.686, *p* < .001). Moreover, the Decision Curve Analysis (DCA) indicated that the OS nomogram yielded a greater net clinical benefit in predicting 5‐year OS in comparison to the 8th TNM system (Supplemental Figure 2).

### 3.4 External validation of the nomogram

The calibration curves for 3‐ and 5‐year OS fitted closely across the external validation cohorts (Figure 2C,D for the pre‐operative nomogram validation; Figure 3C,D for the post‐operative nomogram validation).

The preoperative nomogram similarly demonstrated its efficacy in predicting OS, displaying C‐indices of 0.615 (0.552–0.679) in the validation cohort. It maintained its capacity to accurately categorize patients into distinct prognostic groups within both cohorts (with respective 5‐year OS rates of 45.0%, 29.4%, 26.9%, and 5.3% for the four subgroups in the validation cohort; *p* < .001, Supplemental Figure 1C).

The postoperative nomogram similarly demonstrated precision, displaying C‐indices of 0.719 (0.665–0.773) and exhibiting commendable net clinical benefit in the validation cohort. These values were also superior to those of the 8th TNM system (0.719 vs. 0.686, *p* < .001). The nomogram correctly stratified patients into distinct prognostic subgroups, yielding 5‐year OS rates of 47.8%, 36.4%, 14.6%, and 6.9% within the quartiles of the validation cohort (*p* < .001, Supplemental Figure 1D).

## 4 DISCUSSION

In this study, we formulated two nomograms designed to forecast the extended‐term prognosis for GBC patients who underwent resection. These nomograms displayed precision in prognostic prediction, evident from the congruence between the predictions and actual data demonstrated in the calibration curves. Moreover, the nomograms exhibited consistent performance during external validation. The accurate stratification facilitated by these nomograms could guide postoperative monitoring, facilitate targeted therapy planning, and inform the design of randomized controlled trials. Additionally, the decision curve analysis (DCA) indicated that the postoperative nomogram outperformed the 8th TNM system in terms of net clinical benefit within clinical applications.

We have introduced a predictive nomogram derived from pre‐operative data. The C‐indices of this pre‐operative nomogram for predicting OS ranged from 0.615 to 0.661 in the two cohorts. Apart from surgical resection, alternative treatments for GBC are also accessible, contingent on tumor stage and technical feasibility.^14^ This pre‐operative nomogram offers crucial insights, potentially guiding the consideration of more suitable treatment options for patients projected to have unfavorable post‐operative survival outcomes as indicated by the nomogram.

The postoperative nomogram demonstrated enhanced predictive efficacy, only slightly surpassing the C‐index of the pre‐operative nomogram (0.778 vs. 0.719). The model's precision can aid in tailoring appropriate subsequent therapies for GBC patients prone to unfavorable prognostic outcomes. Additionally, it facilitates streamlined communication between clinicians and patients when determining postoperative treatment strategies. This heightened predictive capability primarily stems from the model's amalgamation of gallbladder physiology, utilization of the strengths of the latest staging system, including lymph node metastasis definition, and integration of various prognostic factors.

An element integrated into the postoperative nomogram but not present in other existing staging systems was the status of the surgical resection margin. Extensive resection is considered imperative for disease eradication. Attaining R0 resection holds significance in determining prolonged survival, with corresponding 5‐year survival rates ranging from 36% to 38%.^5^, ^19^, ^20^ Nonetheless, the efficacy of surgery for advanced GBC remains a subject of debate. Earlier investigations suggested that proactive surgical intervention could enhance survival outcomes for individuals with advanced GBC.^21^, ^22^, ^23^ Conversely, some researchers indicated that tumor biology and stage, rather than the extent of resection, played a more pivotal role in long‐term survival. Addressing these concerns necessitates further prospective studies.

The postoperative nomogram also encompassed lymph node metastasis as a crucial prognostic determinant.^24^ Prior research have underscored that positive regional lymph nodes stand as a significant adverse prognostic indicator for GBC post‐resection.^5^, ^25^ Furthermore, the count of positive lymph nodes autonomously governs the notable prognosis for GBC.^26^ Additionally, the log odds of the number of metastatic lymph nodes and the lymph node ratio have been explored as means to gauge the prognostic impact of lymph node status in GBC patients.^25^ In this investigation, nodal involvement was categorized into two stages based on the 8th AJCC stage system. In conclusion, considering the elevated incidence of nodal involvement in GBC patients undergoing surgical resection, the execution of regional lymphadenectomies is imperative to achieve R0 resection. Our study encompassed regional lymph nodes situated on the hepatoduodenal ligament, hepatic artery, and in the vicinity of the pancreatic head.

The postoperative nomogram also integrated tumor location as a contributing factor. Yang et al. have previously highlighted that gallbladder neck tumors, instead of jaundice, which notably curtails the prospects of radical resection, can independently forecast unfavorable outcomes for GBC patients.^13^ This could potentially be elucidated by the presence of a multitude of organs and structures in the gallbladder neck vicinity, including the contiguous bile duct, portal vein, liver, duodenum, and colon, which may demonstrate early tumor spread, thereby complicating resection and radiotherapy approaches.^27^ Hence, undertaking resection of the extrahepatic bile duct proves valuable for patients afflicted with gallbladder neck tumors.

This nomogram encompassed preoperative clinicopathological parameters, including CA 19‐9.^28^ Preoperative CA19‐9 levels hold significant clinical relevance for early diagnosis. Nonetheless, the precise threshold of CA19‐9 remains to be ascertained. Eun and colleagues demonstrated that a preoperative CA19‐9 level of 37 U/mL or higher stood as an independent prognostic factor influencing survival rates.^29^ Furthermore, elevated preoperative CA19‐9 and CEA levels have been correlated with an unfavorable prognosis in GBC patients subjected to resection.^30^ The integration of CA19‐9 in the nomogram could potentially enhance its predictive accuracy compared to the conventional AJCC system.

Several constraints warrant consideration in this study. Notably, the potential for treatment selection bias could have arisen due to the utilization of retrospective data. Additionally, given that the objective of this study was to formulate a prognostic model centered on resection, the possibility remains that alternative strategies could be more fitting for certain patients within the context of Precision Medicine era.

In summary, two prognostic nomograms for predicting long‐term survival in GBC patients after surgical resection have been developed using pre‐ and post‐operative data. These nomograms demonstrated strong performance. Further external investigations are essential to validate the robustness of these nomograms.

## AUTHOR CONTRIBUTIONS

**Shilei Bai**: Data curation (equal); formal analysis (equal); methodology (equal); writing–original draft (equal). **Pinghua Yang**: Data curation (equal); formal analysis (equal). **Jiliang Qiu**: Data curation (equal); methodology (equal). **Jie Wang**: Methodology (equal); validation (equal). **Liu Liu**: Data curation (equal); methodology (equal). **Chunyan Wang**: Data curation (equal). **Huifeng Wang**: Data curation (equal). **Zhijian Wen**: Data curation (equal). **Baohua Zhang**: Writing–review and editing (equal); project administration (lead); final approval of manuscript: All authors have read and approved the manuscript.

## FUNDING INFORMATION

This study was funded by Natural Science Foundation of Shanghai (81372674) and the Project of Shanghai Shenkang Hospital Development Center (SHDC12016127).

## CONFLICT OF INTEREST STATEMENT

The authors have stated explicitly that there are no conflicts of interest in connection with this article.

## ETHICS STATEMENT

Retrospective review and collection of patient data were approved by the Eastern Hepatobiliary Surgery Hospital and Sun Yat‐Sen University Cancer Center ethics committee. No further ethical approval was required for this retrospective study.

## Supporting information


**Supplemental Table 1.** Types of resection performed in the gallbladder cancer patients.
Supplemental Table 2. Postoperative morbidity of patients between training cohort and validation cohort.
Supplemental Table 3. Univariable analysis of OS based on the preoperative data in the training cohort.
Supplemental Table 4. Univariable analysis of OS based on the postoperative data in the training cohort.

**Supplemental Figure 1.** **Kaplan–Meier curves of OS according to quartiles stratified by the nomogram scores.**
A: The curves by the pre‐operative nomogram in the training cohort.
B: The curves by the pre‐operative nomogram in the external validation cohort.
C: The curves by the post‐operative nomogram in the training cohort.
D: The curves by the post‐operative nomogram in the external validation cohort.

**Supplemental Figure 2.** **The decision curve analysis of the nomogram and conventional criteria.** The *y*‐axis represents net benefits and the *x*‐axis represents threshold probabilities.
The pre‐operative nomogram in the training cohort.
The postoperative nomogram compared with the AJCC 8th system in the training cohort.
The preoperative nomogram in the external validation cohort.
The post‐operative nomogram compared with the AJCC 8th system in the external validation cohort.

## Data Availability

Data will be made available on request.
