# Supplementary material for: Nomograms to predict long‐term survival for patients with gallbladder carcinoma after resection
Source: Cancer Rep (Hoboken). 2024 Mar 5;7(3):e1991. doi: 10.1002/cnr2.1991 (PMC10913079; doi:10.1002/cnr2.1991)
Supplement: Supplementary file 1 — Supplemental Table 1. Types of resection performed in the gallbladder cancer patients. Supplemental Table 2. Postoperative morbidity of patients between training cohort and validation cohort. Supplemental Table 3. Univariable analysis of OS based on the preoperative data in the training cohort. Supplemental Table 4. Univariable analysis of OS based on the postoperative data in the training cohort. [file CNR2-7-e1991-s003.doc]

Supplemental Table 1. Types of resection performed in the gallbladder cancer patients.

| **Treatment** | **Number (Percentage)** | |
| --- | --- | --- |
|  | **Training cohort (n= 378)** | **Validation cohort (n = 108)** |
| **Only gallbladder resection** | 19(5.0%) | 5(4.6%) |
| **Extent of liver resection** |  |  |
| Anatomical segments IVb/V | 106(28.0%) | 38(35.2%) |
| Gallbladder bed | 238(63.0%) | 62(57.4%) |
| Major hepatectomy(>3 segments) | 23(6.1%) | 5(4.6%) |
| Left hepatectomy | 5(1.3%) | 2(1.9%) |
| Trisectionectomy | 4 (1.1%) | 1(0.9%) |
| **Other procedures** |  |  |
| Lymphadenectomy | 378(100%) | 108(100%) |
| Portal vein resection | 3(0.8%) | 1(0.9%) |
| Hepatic artery resection | 6(1.6%) | 1(0.9%) |
| Common bile duct resection | 104(27.5%) | 32 (29.6%) |
| **Adjacent organ resection** |  |  |
| Colectomy | 4(1.1%) | 2(1.9%) |
| Gastric resection | 26(6.9%) | 6(5.6%) |
| Pancreaticoduodenectomy | 3(0.8%) | 1(0.9%) |
| Stomach/duodenum resection | 7(1.9%) | 2(1.8%) |

**Supplemental Table 2. Postoperative morbidity of patients between training cohort and validation cohort.**

| **Complication** | **Training cohort**  **(n= 378)** | **Validation cohort**  **(n = 108)** | ***P* value** |
| --- | --- | --- | --- |
| Wound infection | 18(4.8%) | 9(8.3%) | 0.153 |
| Sepsis/multi-organ failure | 11(7.7%) | 2(1.9%) | 0.742 |
| Abdominal hemorrhage | 8(2.1%) | 1(0.9%) | 0.691 |
| Ileus | 9(2.4%) | 1(0.9%) | 0.699 |
| Bile leak/bile duct obstruction | 32(8.5%) | 6(5.6%) | 0.417 |
| Cholangitis | 11(2.9%) | 1(0.9%) | 0.479 |
| Pneumonia | 20(5.3%) | 2(1.9%) | 0.188 |
| Pleural effusion | 63(16.7%) | 14(13.0%) | 0.455 |
| Pneumothorax | 8(2.1%) | 4(3.7%) | 0.312 |
| Respiratory insufficiency | 23(6.1%) | 5(4.6%) | 0.815 |
| Pulmonary embolism | 3(0.8%) | 2(1.9%) | 0.309 |
| Cardiac diseases | 7(1.9%) | 4(3.7%) | 0.273 |
| Cerebrovascular accident | 13(3.4%) | 2(1.9%) | 0.539 |
| Renal failure | 15(4.0%) | 3(2.8%) | 0.775 |
| Others | 12(3.2%) | 6(5.6%) | 0.253 |
| Total | 124 (32.8%) | 38(35.2%) | 0.645 |
| **Grade of complications*** |  |  |  |
| I-III | 99(26.2%) | 27(25.0%) | 0.901 |
| IV | 18(4.8%) | 7(6.5%) | 0.932 |
| V | 9(2.4%) | 4(3.7%) | 0.496 |

* based on the Clavien-Dindo classification for surgical complication. Grade V complication indicates surgical mortality.

**Supplemental Table 3. Univariable analysis of OS based on the preoperative data in the training cohort**

| **Variable** | **OS** | | | |
| --- | --- | --- | --- | --- |
|  | **HR** | **95.0% CI** | | ***P* value** |
| **Age**, years | 1.006 | 0.995 | 1.017 | 0.294 |
| **Sex**, femal/male | 1.097 | 0.866 | 1.393 | 0.442 |
| **Hypertension**, absence/presence | 1.024 | 0.768 | 1.366 | 0.871 |
| **Diabetes mellitus**, absence/presence | 0.986 | 0.604 | 1.611 | 0.954 |
| **Body mass index**, kg/m^2^ | 0.963 | 0.926 | 1.002 | 0.061 |
| **Associated gallbladder disease**, absence/presence | 1.107 | 0.878 | 1.395 | 0.392 |
| **Jaundice**, presence/absence | 2.121 | 1.641 | 2.736 | <0.001 |
| **TBIL**, μmol/L | 1.002 | 1.001 | 1.003 | <0.001 |
| **ALT**, U/L | 1.001 | 0.994 | 1.002 | 0.061 |
| **CEA**, ng/ml | 1.001 | 0.998 | 1.001 | 0.063 |
| **CA 19-9**, U/ml |  |  |  |  |
| 37-1000 vs ≤37 | 1.468 | 1.121 | 1.541 | 0.005 |
| ≥1000 vs ≤37 | 2.174 | 1.922 | 3.068 | <0.001 |
| **Tumor location(I)** ^†^, neck/fundus or body | 2.141 | 1.677 | 2.731 | <0.001 |
| **Liver or/and Adjacent organ invasion (I)**, yes/no | 1.779 | 1.411 | 2.244 | <0.001 |
| **Abnormal lymph node(I)**, yes/no | 2.213 | 1.655 | 2.959 | <0.001 |

TBIL, total bilirubin; ALT, alanine aminotransferase; CEA: carcinoembryonic antigen; CA19-9: cancer antigen 19-9

^†^ (I): imaging studies;

**Supplemental Table 4. Univariable analysis of OS based on the post**operative data in the training cohort

| **Variable** | **OS** | | | |
| --- | --- | --- | --- | --- |
|  | **HR** | **95.0% CI** | | ***P* value** |
| **Age**, years | 1.006 | 0.995 | 1.017 | 0.294 |
| **Sex**, femal/male | 1.097 | 0.866 | 1.393 | 0.442 |
| **Hypertension**, absence/presence | 1.024 | 0.768 | 1.366 | 0.871 |
| **Diabetes mellitus**, absence/presence | 0.986 | 0.604 | 1.611 | 0.954 |
| **Body mass index**, kg/m^2^ | 0.963 | 0.926 | 1.002 | 0.061 |
| **Associated gallbladder disease**, absence/presence | 1.107 | .878 | 1.395 | 0.392 |
| **Jaundice**, presence/absence | 2.121 | 1.641 | 2.736 | <0.001 |
| **TBIL**, μmol/L | 1.002 | 1.001 | 1.003 | <0.001 |
| **ALT**, U/L | 1.001 | 0.994 | 1.002 | 0.061 |
| **CEA**, ng/ml | 1.001 | 0.998 | 1.001 | 0.063 |
| **CA 19-9**, U/ml |  |  |  |  |
| 37-1000 vs ≤37 | 1.468 | 1.121 | 1.541 | 0.005 |
| ≥1000 vs ≤37 | 2.174 | 1.922 | 3.068 | <0.001 |
| **Extrahepatic bile duct resection**, yes/no | 1.715 | 1.332 | 2.204 | <0.001 |
| **Intraoperative blood transfusion**, yes/no | 1.521 | 1.126 | 2.054 | 0.006 |
| **Resection**, R1/R0 | 4.801 | 3.704 | 6.222 | <0.001 |
| **Tumor location(P)** ^†^, neck/fundus or body | 2.721 | 2.118 | 3.496 | <0.001 |
| **pT category**^*^ |  |  |  |  |
| T2 vs. T1 | 2.302 | 1.419 | 2.556 | 0.001 |
| T3 vs. T1 | 4.088 | 3.953 | 6.536 | <0.001 |
| T4 vs. T1 | 7.235 | 3.734 | 13.241 | <0.001 |
| **pN category**^*^ |  |  |  |  |
| N1 vs. N0 | 3.828 | 2.970 | 3.881 | <0.001 |
| N2 vs. N0 | 6.018 | 4.934 | 9.331 | <0.001 |
| **Tumor differentiation**, poor/well or moderate | 1.927 | 1.453 | 2.555 | <0.001 |

TBIL, total bilirubin; ALT, alanine aminotransferase; CEA: carcinoembryonic antigen; CA19-9: cancer antigen 19-9

^†^ (P): postoperative pathological examinations

* pT category and pN category were defined according to the 8th edition of the AJCC staging system.
