# Supplementary material for: Nomograms to predict long‐term survival for patients with gallbladder carcinoma after resection
Source: Cancer Rep (Hoboken). 2024 Mar 5;7(3):e1991. doi: 10.1002/cnr2.1991 (PMC10913079; doi:10.1002/cnr2.1991)
Supplement: Supplementary file 2 — Supplemental Figure 1. Kaplan–Meier curves of OS according to quartiles stratified by the nomogram scores. A: The curves by the pre‐operative nomogram in the training cohort. B: The curves by the pre‐operative nomogram in the external validation cohort. C: The curves by the post‐operative nomogram in the training cohort. D: The curves by the post‐operative nomogram in the external validation cohort. [file CNR2-7-e1991-s001.pdf]

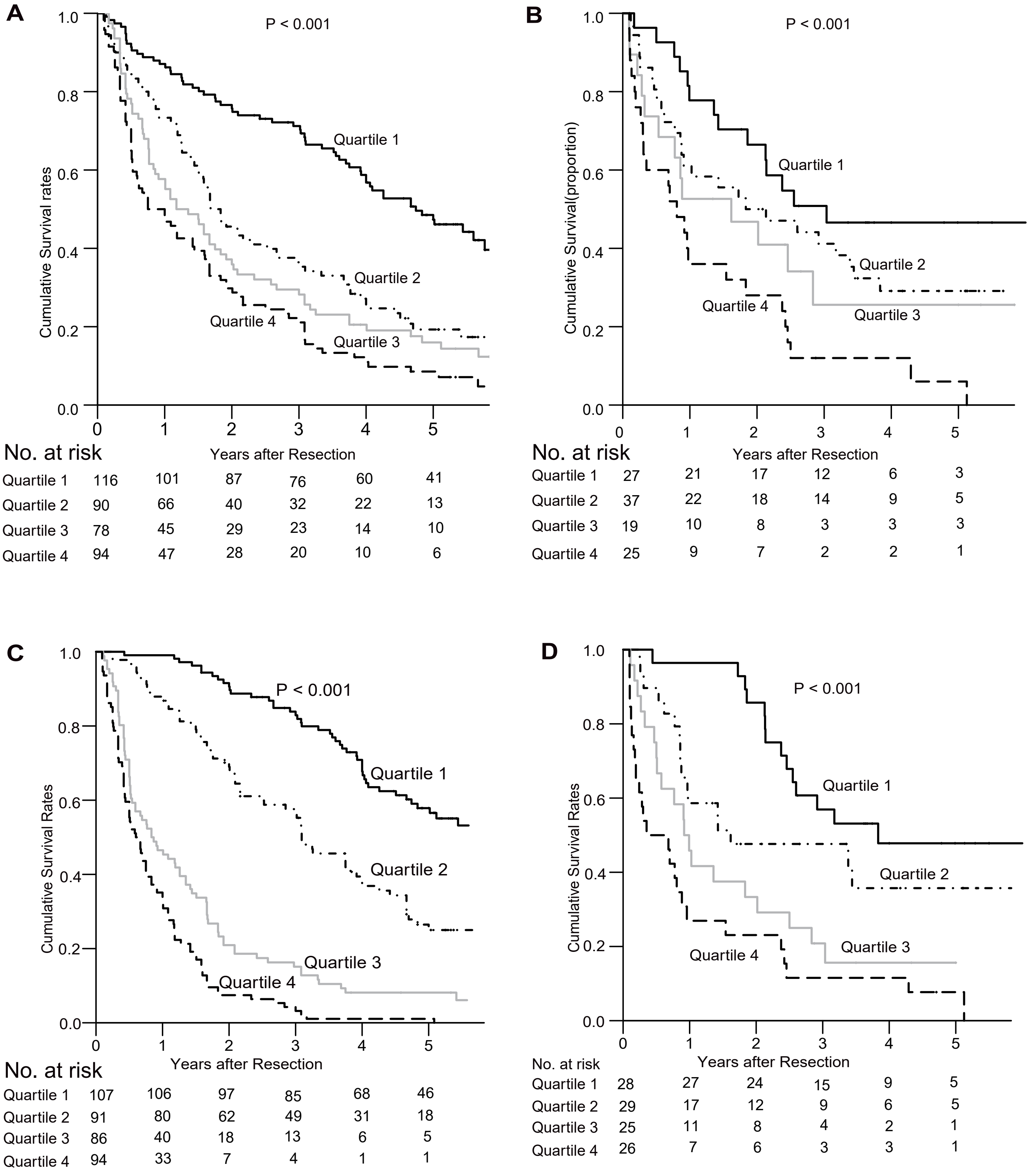

Supplemental Figure 1. Kaplan-Meier curves of OS according to quartiles stratified by the nomogram scores.
A: The curves by the pre-operative nomogram in the training cohort.
B: The curves by the pre-operative nomogram in the external validation cohort.
C: The curves by the post-operative nomogram in the training cohort.
D: The curves by the post-operative nomogram in the external validation cohort.
