# Supplementary material for: Nomograms to predict long‐term survival for patients with gallbladder carcinoma after resection
Source: Cancer Rep (Hoboken). 2024 Mar 5;7(3):e1991. doi: 10.1002/cnr2.1991 (PMC10913079; doi:10.1002/cnr2.1991)
Supplement: Supplementary file 3 — Supplemental Figure 2. The decision curve analysis of the nomogram and conventional criteria. The y‐axis represents net benefits and the x‐axis represents threshold probabilities. The pre‐operative nomogram in the training cohort. The postoperative nomogram compared with the AJCC 8th system in the training cohort. The preoperative nomogram in the external validation cohort. The post‐operative nomogram compared with the AJCC 8th system in the external validation cohort. [file CNR2-7-e1991-s002.pdf]

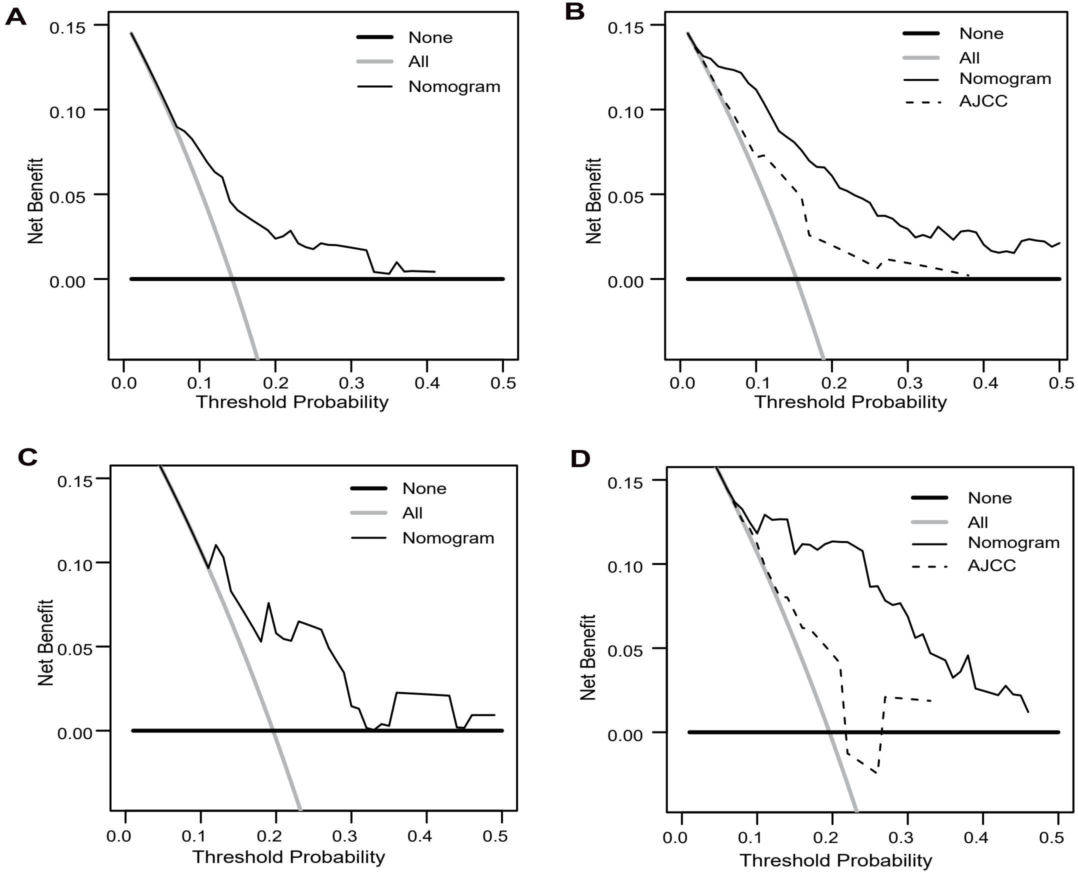

Supplemental Figure 2. The decision curve analysis of the nomogram and conventional criteria.
The y-axis represents net benefits and the x-axis represents threshold probabilities..
A.The pre-operative nomogram in the training cohort.
B.The postoperative nomogram compared with the AJCC 8th system in the training cohort.
C.The preoperative nomogram in the external validation cohort.
D.The post-operative nomogram compared with the AJCC 8th system in the external validation cohort.
